# *Clostridium difficile* Peritonitis: An Emerging Infection in Peritoneal Dialysis Patients

**DOI:** 10.1155/2018/3537283

**Published:** 2018-09-19

**Authors:** Saira Chaughtai, Bhavika Gandhi, Zeeshan Chaughtai, Dana Tarina, Mohammad A. Hossain, Arif Asif

**Affiliations:** Department of Medicine, Jersey Shore University Medical Center, Hackensack Meridian Health, 1945 Route 33, Neptune, NJ 07753, USA

## Abstract

Recently, the incidence of *Clostridium difficile-* (*C. difficile-*) associated infection has increased significantly in hospital and ambulatory care settings in parallel to the increasing use of inappropriate antibiotics. According to the CDC, approximately 83,000 patients who developed *C*. *difficile* experienced at least one recurrence and 29,000 died within 30 days of the initial diagnosis. Patients on dialysis (particularly peritoneal dialysis) are predisposed to this infection due to an inherent immunocompromised state and transmural translocation of the bacteria due to the close association of gastrointestinal tract and peritoneal cavity. *C*. *difficile* infection in peritoneal dialysis patients is problematic from two aspects: (1) because dialysis patients are immunocompromised, the infection can be devastating and (2) infection directly interferes with their renal replacement therapy. In this article, we present a case of peritoneal dialysis (PD)-related peritonitis caused by *C. difficile*-associated diarrhea and colitis. In this patient, the peritonitis was caused by transmural translocation of the enteric bacteria. While the peritoneal fluid culture did not grow the organism (possibly because of prior empiric broad-spectrum antibiotics use), the positive PCR on stool analysis suggested *C. difficile*-related peritonitis, along with the rapid clinical improvement induced by *C*. *difficile*-directed therapy (metronidazole) and discontinuation of broad-spectrum antibiotics. The patient was successfully treated with metronidazole without PD catheter removal. *C*. *difficile* infection is common and frequently internists are the first contact with such patients. This article highlights *C*. *difficile* infection in a PD patient and raises awareness of this infection in dialysis patients.

## 1. Introduction

Recent data have emphasized that the incidence of *C*. *difficile* is on the rise because of multiple factors (use of inappropriate antibiotics, emergence of new virulent *C. difficile* strains, increased median age of the population, and multiple comorbidities). Dialysis patients are immunocompromised and hence are vulnerable to infection and sepsis from *C*. *difficile* colitis. There have been cases with *C*. *difficile* peritonitis with negative cultures from the PD catheter. In general, a positive *C*. *difficile* toxin assay and clinical resolution of symptoms with treatment are the only clue to diagnosis. In this report, we present a case of *C. difficile* peritonitis with its diagnosis and management strategy.

## 2. Case

An 85-year-old black woman was admitted to the hospital with complaints of crampy abdominal pain, vomiting, and several episodes of watery diarrhea for two days. Her past medical history included end-stage renal disease (on continuous cycling peritoneal dialysis), congestive heart failure, atrial fibrillation, chronic obstructive pulmonary disease, and cervical cancer (status after total abdominal hysterectomy). She was started on peritoneal dialysis three months ago for her end-stage renal disease during a hospital admission for congestive heart failure. She was discharged to rehab, where she stayed for a month. Her last home dialysis session was a day prior to the onset of symptoms. She denied any fever, bloody stool, outside food ingestion, any sick contact, and recent travel history. On physical examination, her vital signs revealed a pulse rate of 76 beats per minute, blood pressure of 121/70 mmHg, respiratory rate of 16 breaths per minute, and a temperature of 99°F. Abdominal examination revealed nondistended abdomen with diffuse tenderness on palpation and hyperactive bowel sounds with voluntary guarding; however, dialysis catheter was intact with no surrounding erythema or purulent discharge. Initial laboratory analysis showed WBC of 13,400/*μ*L, neutrophils of 88.5%, hemoglobin of 11 g/dl, and serum albumin of 2.9 mg/dl. Peritoneal fluid analysis revealed white blood cell count in peritoneal effluent of 8359/*μ*L, with 93% neutrophil predominance. Gram strain did not show any microorganisms. Given the overall clinical status, peritonitis was suspected, and she received empiric vancomycin and ceftazidime. However, stool *C. difficile* toxin B assay returned positive. She was concurrently started on oral metronidazole. Within 24 hours, there was improvement in the clinical symptoms, and her broad-spectrum antibiotics were discontinued after a couple of days. The patient was diagnosed with PD-related peritonitis accompanying *C. difficile*-associated diarrhea, with continuation of oral metronidazole. Repeated effluent white blood cell count the day after starting metronidazole decreased to 133/*μ*L, with complete resolution of peripheral leukocytosis. Surgical consultation done on admission concluded that the peritoneal catheter was not the source of infection and, therefore, the catheter was not removed. The patient's symptoms resolved, and she was then discharged on a total of three weeks of metronidazole with continuation of peritoneal dialysis.

## 3. Discussion

Peritonitis is a major complication of PD and is the one that may dissuade end-stage renal disease patients from choosing this form of renal replacement therapy [1]. The incidence of *C*. *difficile* infection has increased significantly for the last few years in the United States [2]. *C*. *difficile* infection has a major economic impact on costs of medical care related to hospital stay and mortality [3, 4]. Because of their immunocompromised state, PD patients are vulnerable to develop this infection. Such infections produce peritonitis by transmural translocation of the bacteria into the peritoneal cavity [5]. The diagnosis for it may only depend on a positive *C*. *difficile* stool toxin assay (PCR), as well as clinical resolution of the peritoneal infection with the start of treatment. In our case, the patient's effluent culture was negative for organisms, but positive stool PCR and clinical resolution with the appropriate treatment for *C. difficile* confirmed the diagnosis.

The disruption of the gastrointestinal barrier allows enteric bacteria to translocate into the peritoneal cavity, which is a major mechanism of peritonitis [5, 6]. Additionally, the presence of dextrose in the PD fluid allows for relatively easy seeding of the peritoneal cavity [5, 6]. Interestingly, culture-negative peritonitis is associated with lower risk of recurrent peritonitis [7]. Clinical presentation of *C. difficile* infection varies. It can range from mild to severe disease and can start as diarrhea/abdominal pain, low to high-grade fever, and leukocytosis. In severe cases, it may progress to hypovolemic shock. It is worth mentioning that acute diarrhea and hypoalbuminemia, which is known to result from the oozing of serum albumin from plasma into the ulcerated colonic mucosa, are good indicators of *C. difficile* intestinal disease [8]. While our patient's albumin was 2.9 mg/dl, low albumin can be seen in PD patients without *C*. *difficile* peritonitis and is not specific for *C. difficile* infection [9]. A definitive diagnosis of *C*. *difficile* colitis can be made by *C*. *difficile* toxin assay [10].

Of note, PD patients with positive peritoneal fluid culture for *C. difficile* have been reported in the literature [11]. In this patient, the *C. difficile* peritonitis was fatal [11]. In contrast, our case was culture-negative in peritoneal fluid, but there was positive stool *C. difficile* PCR, and clinical resolution with metronidazole. It is worth noting that whether the diagnosis is made by a positive stool assay or positive dialysate culture, vancomycin is now proven superior to metronidazole for the initial *C. difficile* diarrhea [12]. Our dialysis patient responded well to metronidazole therapy, as this was the preferred treatment agent at that time. We extended the treatment to three weeks though there are no guidelines or recommendations for treatment of *C. difficile* peritonitis for PD patients in the literature, as complete eradication in a patient with multiple comorbidities was desired.

## 4. Conclusion

*C. difficile* is a common infection today. Because of their immunocompromised status, dialysis patients are vulnerable to acquiring this infection. Clinical presentation, high white blood cell count in the blood and peritoneal effluent, and a positive peritoneal fluid culture or stool PCR are helpful in establishing the diagnosis. Our case was successfully treated with oral metronidazole, which was the first-line therapy at the time. Because *C. difficile* can cause death, heightened awareness, timely diagnosis, and prompt treatment are required.

## References

[1] Mancini A., Todd L. (2018). Inconsistencies in ISPD peritonitis recommendations: 2016 update on prevention and treatment and the ISPD catheter-related infection recommendations: 2017 update. *Peritoneal Dialysis International*.

[2] Khanna S., Pardi D. S., Aronson S. L. (2012). The epidemiology of community-acquired *Clostridium difficile* infection: a population-based study. *American Journal of Gastroenterology*.

[3] Pakyz A., Carroll N. V., Harpe S. E., Oinonen M., Polk R. E. (2011). Economic impact of *Clostridium difficile* infection in a multihospital cohort of academic health centers. *Pharmacotherapy*.

[4] CDC (2015). *Clostridium difficile Infection*.

[5] Singharetnam W., Holley J. L. (1996). Acute treatment of constipation may lead to transmural migration of bacteria resulting in gram-negative, polymicrobial, or fungal peritonitis. *Peritoneal Dialysis International*.

[6] Allen S. D., Connor D. H., Chandler F. W., Schwartz D. A., Manz H. J., Lack E. E., Manz H. (1997). Pig-bel and other necrotizing disorders of the gut involving *Clostridium perfringens*. *Pathology of Infectious Diseases*.

[7] Hamad A., Imail H., Elsayed M. (2018). The epidemiology of acute peritonitis in end-stage renal disease patients on peritoneal dialysis in Qatar: an 8-year follow-up study. *Saudi Journal of Kidney Diseases and Transplantation*.

[8] Bartlett J. G., Feldman M., Freidman L. S., Sleisenger M. H. (2002). Pseudomembranous enterocolitis and antibiotic-associated diarrhea. *Sleisenger and Fordtran’s Gastrointestinal and Liver Disease Pathophysiology/Diagnosis/Management*.

[9] Arikan T., Unal A., Kocyigit I., Yurci A., Oymak O. (2014). Peritoneal dialysis-related peritonitis triggered by *Clostridium difficile*-associated colitis. *Peritoneal Dialysis International*.

[10] Bharti S., Malhotra P., Juretschko S. (2015). Successful treatment of peritoneal dialysis catheter-related polymicrobial peritonitis involving *Clostridium difficile*. *Journal of Clinical Microbiology*.

[11] Laroche M. C., Alfa M. J., Harding G. K. (1997). Isolation of toxigenic *Clostridium difficile* from dialysate fluid in a fatal case of chronic ambulatory peritoneal dialysis-related peritonitis. *Clinical Infectious Diseases*.

[12] Marco F., Giusy T., Federica I. (2018). Risk factors for recurrence in patients with *Clostridium difficile* infection due to 027 and non-027 ribotype. *Clinical Microbiology and Infection*.

